# Evolution of vertebrate retinal photoreception

**DOI:** 10.1098/rstb.2009.0102

**Published:** 2009-10-12

**Authors:** Trevor D. Lamb

**Affiliations:** 1 ARC Centre of Excellence in Vision Science, The Australian National University, Canberra ACT 0200, Australia; 2 Eccles Institute of Neuroscience, The Australian National University, Canberra ACT 0200, Australia; 3 John Curtin School of Medical Research, The Australian National University, Canberra ACT 0200, Australia

**Keywords:** evolution, vertebrate photoreceptor, vertebrate retina, rhabdomeric photoreceptor, opsins, eyes

## Abstract

Recent findings shed light on the steps underlying the evolution of vertebrate photoreceptors and retina. Vertebrate ciliary photoreceptors are not as wholly distinct from invertebrate rhabdomeric photoreceptors as is sometimes thought. Recent information on the phylogenies of ciliary and rhabdomeric opsins has helped in constructing the likely routes followed during evolution. Clues to the factors that led the early vertebrate retina to become invaginated can be obtained by combining recent knowledge about the origin of the pathway for dark re-isomerization of retinoids with knowledge of the inability of ciliary opsins to undergo photoreversal, along with consideration of the constraints imposed under the very low light levels in the deep ocean. Investigation of the origin of cell classes in the vertebrate retina provides support for the notion that cones, rods and bipolar cells all originated from a primordial ciliary photoreceptor, whereas ganglion cells, amacrine cells and horizontal cells all originated from rhabdomeric photoreceptors. Knowledge of the molecular differences between cones and rods, together with knowledge of the scotopic signalling pathway, provides an understanding of the evolution of rods and of the rods' retinal circuitry. Accordingly, it has been possible to propose a plausible scenario for the sequence of evolutionary steps that led to the emergence of vertebrate photoreceptors and retina.

## 1. Introduction

The purpose of this review is to consider how it may have been that photoreception in our eyes evolved. The term ‘photoreception’ will be interpreted broadly, to include the nature of the photoreceptor cells, of their opsin photopigments, and of their signalling cascades, as well as the structure of the retina, and the processing of the signals from the photoreceptors by subsequent neurons in the vertebrate retina.

The emphasis will be on events that occurred in our own ancestors; i.e. in the line leading to mammals, as illustrated in the evolutionary trees in several figures in this Theme Issue (e.g. Larhammar *et al*. 2009; figure 2). The consideration of this evolutionary line will run from the primitive bilaterally symmetric organisms that predated the divergence of protostomes and deuterostomes, through the emergence of early chordates, through the Cambrian explosion when craniates and vertebrates appeared, and through to the near-perfection of vertebrate retinal photoreception that had been reached by the time that jawed vertebrates appeared.

The evidence and arguments relating to a number of major advances that occurred at successive times during vertebrate evolution will be presented in separate sections. It must be emphasized, though, that uncertainties abound, and that a good deal of speculation is involved. Finally, a scenario will be described for the overall sequence and likely timing of the principal events that led to the evolution of our retina and its photoreceptors.

## 2. Origin of vertebrate retinal photoreceptors and opsins

Until quite recently, it had generally been thought that the photoreceptor cells of most invertebrates (protostomes) were rhabdomeric, whereas the photoreceptor cells of vertebrates were ciliary (see Eakin 1965). However, over the last decade it has become clear that there are numerous examples where a given vertebrate or invertebrate species may contain both ciliary and rhabdomeric photoreceptors (Arendt & Wittbrodt 2001; Arendt 2003; Arendt *et al*. 2004). The human genome contains both ciliary opsins (*c*-opsins) and melanopsin, the latter of which is clearly a member of the rhabdomeric opsin (*r*-opsin) family.

It is now widely agreed that, prior to the divergence of protostomes and deuterostomes, some 600 Ma, bilaterally symmetric animals already possessed at least these two classes of photoreceptor: rhabdomeric and ciliary. The primordial rhabdomeric photoreceptor contained an *r*-opsin, and is thought to have coupled to a G_q_ G-protein cascade that used phospholipase C (PLC) as the effector protein. The primordial ciliary photoreceptor contained a *c*-opsin, and is thought to have coupled to a precursor of the G_t_ (transducin) G-protein cascade that uses PDE6 (the cGMP phosphodiesterase) as the effector protein.

Very recently it has been shown that photoreceptors in the eyes of the cnidarian box jellyfish share many features in common with vertebrate ciliary photoreceptors (Koyanagi *et al*. 2008; Kozmik *et al*. 2008), even though cnidaria diverged from the ancestors of bilateral animals long before the protostome/deuterostome split; the interpretation of these findings will be discussed in §2*c* below.

### (a) Vertebrate ciliary photoreceptors

**Figure 1.**
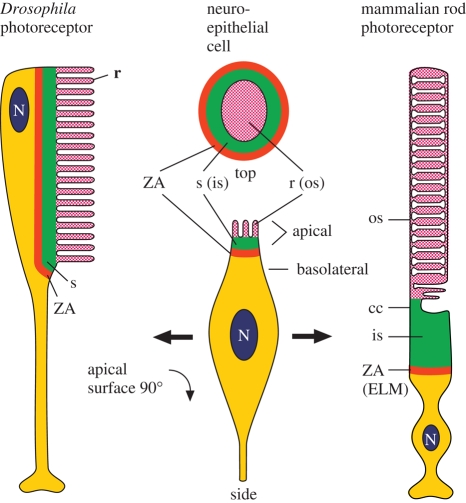
Conservation of cell polarity and topology between *Drosophila* rhabdomeric and mammalian ciliary photoreceptor cells (see text). r, rhabdome; s, fly stalk; ZA, zonula adherens; os, outer segment; cc, connecting cilium; is, inner segment; ELM, external limiting membrane; N, nucleus. Adapted with permission from Ready & Tepass (2004).

Despite the major differences between them, the rhabdomeric photoreceptors of protostomes and the ciliary photoreceptors of the vertebrate retina share a number of structural homologies, as illustrated in figure 1 (Ready & Tepass 2004). The figure in the middle represents an immature (or perhaps a primitive) generic photoreceptor cell with bipolar morphology. The red band indicates a circumferential zonula adherens (ZA) junctional complex that delimits apical membrane from basolateral membrane; in the vertebrate retina this forms the external limiting membrane. Within the apical region, two subdomains develop. Most distally (pink), an enormous expansion of membrane surface area occurs, either in the form of microvilli in the rhabdome (r), or as a stack of flattened discs in the mammalian outer segment (os). This photoreceptive region is separated from the ZA by a supportive subdomain (green): in *Drosophila*, the fly stalk (s), or in vertebrates, the photoreceptor inner segment (is) containing the 9 + 0 structure of the connecting cilium (cc). In rhabdomeric photoreceptors, the apical end of the cell is rotated by 90°.

From this perspective, rhabdomeric photoreceptors and vertebrate ciliary photoreceptors appear structurally homologous. If these morphological similarities arise because both classes of photoreceptor evolved from a common precursor cell, then the two classes can be considered ‘sister’ cells according to the terminology of Arendt (2003).

As has been described recently by Lamb *et al*. (2007), the ciliary photoreceptors of chordates exhibit properties that are consistent with the notion that there has been a gradual transition in morphology, from a simple structure (not unlike that depicted in the middle panel of figure 1) in basal chordates, to the specialized and compartmentalized structure of cones and rods in jawed vertebrates. Combining the insights from Ready & Tepass (2004) and Lamb *et al*. (2007), one may plausibly trace the sequences of changes in morphology that occurred both during the evolution of rhabdomeric photoreceptors and during the evolution of our own photoreceptors.

It seems likely that a cilium was already present in the common precursor of both classes of photoreceptors (not shown in figure 1, middle), though it is not clear whether surface extensions would have been present. In the rhabdomeric line the cilium was lost and the apical membrane extended in the form of microvilli. At some stage, the apical end of the cell underwent a 90° rotation aligning the microvilli at right angles to the direction of incident light (figure 1, left). In the ciliary line, the primary cilium was retained and the apical membrane extended from it, possibly originally in the form of microvilli (as is often seen in the photoreceptors of extant cnidarians and non-vertebrate chordates). During chordate evolution, the membrane extensions became flattened (instead of tubular), in due course becoming organized as a longitudinal stack of sacs, and eventually discs (figure 1, right).

### (b) Vertebrate ciliary opsins

A major advance in the understanding of the evolution of vertebrate retinal opsins was achieved when Okano *et al*. (1992) showed that the rod opsin had evolved from one of the four pre-existing cone opsins. Numerous subsequent studies have extended our knowledge of the phylogenetic relationship between opsins in a vast range of organisms; see, for example, Yokoyama (2000); Arendt & Wittbrodt (2001); Terakita (2005); Suga *et al*. (2008); Shichida & Matsuyama (2009). As a result we can now trace the ancestry of animal opsins both in great detail and far back into distant times.

**Figure 2.**
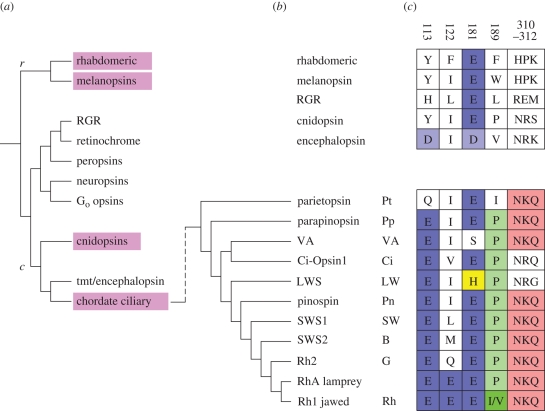
Ciliary opsins. (*a*) Simplified phylogenetic tree of animal opsins based on Suga *et al*. (2008); *r* denotes (rhabdomeric) *r*-opsins; *c* denotes (ciliary) *c*-opsins. Mauve background indicates those opsin classes that are discussed in the text. (*b*) Phylogenetic relationship of chordate ciliary opsins, summarizing a consensus view from a number of studies (including Okano *et al*. 1992, 1994; Yokoyama 2000; Kusakabe *et al*. 2001; Collin *et al*. 2003; Carleton *et al*. 2005; Kozmik *et al*. 2008; Peirson *et al*. 2009; Shichida & Matsuyama 2009). (*c*) Notable amino acid residues that assist in distinguishing the properties of chordate ciliary opsins. Numbering is according to bovine rhodopsin; coloured shading is simply to aid visualization of groupings.

A phylogenetic tree of animal opsins, based on the recent study by Suga *et al*. (2008), is illustrated in figure 2*a*, with the two main families involved in photoreception denoted as *r*-opsins and *c*-opsins. Between these two groupings is shown a less well understood cluster of opsins that includes the photoisomerases of protostomes and RGR (retinal G protein-coupled receptor) of the vertebrate RPE (retinal pigment epithelium), together with the peropsins of both protostomes and vertebrates, as well as the neuropsins and G_o_-coupled opsins.

The *r*-opsins comprise the rhabdomeric opsins of protostomes together with the melanopsins of chordates, and couple to a G_q_ cascade. The *c*-opsins are always found in ciliated photoreceptor cells, and include the teleost multiple tissue (tmt) opsins and encephalopsins, together with the ciliary opsins of chordate photoreceptors, the latter of which generally couple to a G_t_ cascade. It has recently been discovered that the ‘cnidopsins’ of jellyfish (cnidarians) clade with the *c*-opsins (see §2*c* below). Since cnidarians diverged from bilateral animals long before the protostome/deuterostome split (see fig. 2 of Larhammar *et al*. 2009), it can be concluded that the separate classes of *c*-opsins and *r*-opsins were already present in primitive metazoa prior to the divergence of bilateria and cnidaria.

The branch of chordate ciliary opsins from figure 2*a* has been expanded in figure 2*b*. All five classes of vertebrate retinal opsin genes (*LWS*, *SWS1*, *SWS2*, *Rh2*, *Rh1*) arose relatively recently, from a common precursor, with the rod opsin gene (*Rh1*) having arisen from one of the cone opsin genes, namely *Rh2* (Okano *et al*. 1992; see §5*b*). As reviewed by Nordström *et al*. (2004) and Larhammar *et al*. (2009), these branchings are broadly consistent with two rounds of genome duplication (2R) at the base of the vertebrate lineage, though the exact timing of these two tetraploidizations remains unclear. Lampreys possess five classes of retinal opsin that appear closely homologous to those of jawed vertebrates (Davies *et al*. 2007), whereas tunicates (e.g. *Ciona intestinalis*) have so far only been shown to possess a single copy of a *c*-opsin. It will be of considerable interest to find the opsins of hagfish, and to examine the homologies that they exhibit to vertebrate visual opsins.

Figure 2*c* shows the amino acid residues at several locations (in the numbering of bovine rod opsin) that have been shown by Shichida and colleagues to be important in distinguishing vertebrate visual opsins (reviewed in Imai *et al*. 2005 and Shichida & Matsuyama 2009; see §5*b*). It is clear that the primordial opsin had its counterion for the protonated Schiff base at position 181 (E181), and this site is retained as a negatively charged residue in almost all animal opsins. Chordate ciliary opsins evolved a second negatively charged residue at site 113 (E113), which has been shown to act as the Schiff base counterion for the resting state of vertebrate rhodopsin; thus, the site of the counterion in the ground state has migrated during the evolution of chordate ciliary opsins (Terakita *et al*. 2004).

The likely advantage of the second counterion site for the protonated Schiff base was that it permitted stabilization of a second configuration: E113 could stabilize the ground state (i.e. the resting state that binds 11-*cis*-retinal), while the original E181 could stabilize the active metarhodopsin state(s), improving the performance of the light-activated state, by increasing its ability to interact with the G-protein. Thus, Yan *et al*. (2003) provided evidence suggesting that E181 stabilizes the metarhodopsin I state, and Scheerer *et al*. (2008) provided evidence that it stabilizes the activated metarhodopsin during its interaction with the G-protein. It seems likely, though, that a consequence of the possession of this second counterion site has been the inability of the metarhodopsin state to be photoisomerized back to the ground state; i.e. elimination of the property of photoreversal exhibited by those opsins not possessing the counterion at residue 113 (see §3*a*(i)).

As will be described below, it appears that the loss of photoreversal has had profound consequences for chordate photoreception, in necessitating a separate biochemical pathway for the resynthesis of 11-*cis*-retinal, and thereby leading to the ‘inside-out’ organization of the vertebrate retina/RPE, with the photoreceptors being positioned outermost in the retina.

### (c) Cnidarian opsins and photoreceptors

Cnidaria are the most basal phylum to possess eyes, and it has long been known that cnidarian photoreceptors are ciliated (Eakin & Westfall 1962; Yamasu & Yoshida 1976; Martin 2002). Very recently, it has been reported that photoreception in box jellyfish exhibits remarkable parallels to photoreception in vertebrates. Thus, their opsins (cnidopsins) have been reported to clade with the *c*-opsins (Koyanagi *et al*. 2008; Kozmik *et al*. 2008; Suga *et al*. 2008; figure 2*a*). In addition, it has been reported that signalling components broadly similar to those of the vertebrate phototransduction cascade are present (Kozmik *et al*. 2008), though the G-protein is of the stimulatory (G_s_) kind and light stimulates an increase in cAMP levels (Koyanagi *et al*. 2008).

This evidence, taken together with the knowledge of the very ancient divergence of cnidaria from the bilateral line, has led to the suggestion that the primordial photoreceptor and opsin were of the ciliary class, and that these have been preserved in the vertebrate line; on this basis, the rhabdomeric opsin and photoreceptor would have evolved from ciliary precursors (reviewed in Nilsson 2009). However, Kozmik *et al*. (2008) have listed a number of lines of evidence, including the roles of transcription factors, that argue against a close homology between cnidarian and vertebrate retinal photoreceptors, and that led them to favour the idea that the eyes of vertebrates and box jellyfish arose by independent recruitment of orthologous genes, rather than via common ancestry.

Two further considerations may be relevant. Firstly, although cnidopsins are similar to the *c*-opsins of vertebrates (figure 2*a*), they lack the characteristic E113 residue of all chordate visual opsins (figure 2*c*), that may represent a key feature of the mechanism of chordate phototransduction. Secondly, the topology of the opsin-containing membranes of cnidarian photoreceptors, comprising numerous microvilli (Eakin & Westfall 1962; Yamasu & Yoshida 1976; Martin 2002), resembles that of many invertebrate species, including chordates such as *Amphioxus* and *Ciona*, as distinct from the flattened sacs of vertebrates. Thus, the ciliary photoreceptors and ciliary opsins of cnidaria are remarkably similar to those of the most basal chordates.

### (d) Rhabdomeric opsins and melanopsin

The *r*-opsins employed in rhabdomeric photoreceptors of invertebrates, and (as melanopsin) in intrinsically photosensitive retinal ganglion cells of vertebrates, do not play any role in vertebrate ciliary phototransduction and will not be covered here. The reader is instead referred to Koyanagi & Terakita (2008); Pierson *et al*. (2009); Shichida & Matsuyama (2009) for further information.

## 3. Origin of the vertebrate eyecup

It seems reasonable to speculate that the photosensitive organs of early chordates, some 550 Ma, may have resembled those of extant cephalochordates (e.g. the lancelet, *Amphioxus* or *Branchiostoma*) and tunicates (e.g. *Ciona*, *Amaroucium*). These living species possess simple ocelli, containing a small number of photoreceptor cells together with one or a few pigment cells. Although they sense light, they do not have image-forming vision as we know it, and any directional light sense that they possess is mediated by a fairly small number of photoreceptors that are shielded by dark pigment (see Nilsson 2009 for a review of similar organs in protostomes).

*Branchiostoma* possesses two organs with ciliary photoreceptor cells: the paired ‘frontal eyes’ that have been postulated to be homologues of the vertebrate lateral eyes and, caudal to these, the ‘lamellar body’ that has been postulated to be the homologue of the vertebrate pineal (reviewed in Lacalli 2004). Further caudally, *Branchiostoma* additionally possesses two varieties of rhabdomeric photoreceptor: ‘Joseph cells’ in the dorsal-most layer of the nerve cord, and deeper ‘dorsal ocelli’ in which the rhabdomeric photoreceptor cell is associated with a pigmented cell (Lacalli 2004).

The tadpole-like larva of many tunicates (e.g. *Ciona*, *Amaroucium*) possesses a single ocellus, comprising a single cup-shaped pigment cell, three lens cells and about 30 ciliary photoreceptors (Barnes 1971). The single pigment cell of the ocellus (like that of a nearby gravity-detecting organ) develops from bilateral precursors, where additional (supernumerary) cells die by programmed cell death (Jeffery 2004); it is possible that the ocellus represents one half of an earlier bilateral photoreceptive system (Horie *et al*. 2008). Hence, at present it is not clear whether the ascidian ocellus is a homologue of the vertebrate lateral eye, or a homologue of the vertebrate pineal/parapineal (Kusakabe *et al*. 2001), or both. As discussed below, the pigmented cell is likely to mediate the biochemical reconversion of all-*trans*-retinal to its 11-*cis* isomer, and so this cell might represent a homologue of the vertebrate RPE.

Tunicates have also been reported to possess rhabdomeric photoreceptors. In the siphon eye spots of the adult *Ciona*, Dilly & Wolken (1973) found presumptive photoreceptors with a simple cilium and with microvilli extending from the non-ciliary membrane; this resembles the situation in some protostomes and they referred to the cell as a rhabdomeric photoreceptor. Rhabdomeric photoreceptors have also been reported to exist in the cerebral eyes of salps (Lacalli 2004, p. 150).

### (a) Implications of the nature of the regeneration of 11-*cis*-retinaldehyde

In order to regenerate the bent 11-*cis* isomer of retinaldehyde, so that the visual pigment molecule can again respond to another photon of light, the rhabdomeric photoreceptors of protostomes and the ciliary photoreceptors of vertebrates have adopted fundamentally different approaches. However, an intermediate situation appears to occur in the tunicate *Ciona* (Tsuda *et al*. 2003; Kusakabe *et al*. 2009). Comparison of the three approaches may help to shed light on the factors that led to the evolution of the vertebrate eyecup.

#### (i) Inability of ciliary opsins to undergo photoreversal to the ground state

The photoreceptors of protostomes employ light to regenerate visual pigment, either directly via the photopigment itself, or indirectly via a separate photoisomerase protein. Thus, rhabdomeric opsins are able to regenerate the native rhodopsin state by photoreversal, whereby the stable metarhodopsin is isomerized back to rhodopsin by the absorption of a second photon; the absorbance spectrum peaks at longer wavelengths than rhodopsin's absorbance (e.g. typically in the yellow). In contrast, ciliary opsins have lost this ability to photoreverse, probably in part as a result of the relocation of the counterion site (Terakita *et al*. 2004), from E181 to E113. In cephalopods, a photoisomerase known as retinochrome can take all-*trans*-retinal and photoisomerize it to the 11-*cis* form, for delivery by a binding protein, back to opsin.

In mammalian phototransduction, activation of rhodopsin by a photon triggers the protein molecule to undergo at least one proton translocation as well as the uptake of an additional proton. Yan *et al*. (2003) have proposed that, by the time that metarhodopsin I is formed (within approx. 1 ms of photoisomerization), a proton has been translocated from E181 to E113, so that the counterion for the Schiff base bond changes from being E113 in the ground state to being E181 in metarhodopsin I. From the crystal structure of active bovine opsin, Scheerer *et al*. (2008) report that E181 stabilizes the activated metarhodopsin during its interaction with the G-protein. In the metarhodopsin II state, a proton is taken up from the aqueous environment by E134 (Arnis *et al*. 1994). E134 is part of the E(D)RY motif that is highly conserved across all G-protein-coupled receptors (GPCRs). Knierim *et al*. (2007) reported that this protonation of E134 is a consequence of the rearrangement of helices that occurs during formation of the enzymatically active metarhodopsin II, and presumably it stabilizes this state.

In any case, this series of changes in protein configuration and in the state of protonation apparently renders it impossible for photoreversal to occur. For bovine rhodopsin, the possibilities for photoconversion between different states of the protein molecule were investigated by Arnis & Hofmann (1995). Once the molecule has reached the activated form of metarhodopsin II (which they termed MIIa), they found that it could not be converted back to the ground state by photon absorption.

Thus, the advantage that the basal deuterostome ciliary opsin gained through its counterion relocation (see Terakita *et al*. 2004), of more efficient activation of the G-protein, was counterbalanced by the loss of its former ability to undergo photoreversal.

#### (ii) Dark isomerization of retinoid to the 11-*cis* configuration

The vertebrate retina employs a complicated cycle to resynthesize the 11-*cis* isomer of retinaldehyde in darkness (reviewed in Lamb & Pugh 2004; Travis *et al*. 2007; Kusakabe *et al*. 2009). The visual pigment in the ciliary photoreceptor covalently binds 11-*cis*-retinaldehyde. This chromophore is isomerized by the absorption of a photon to its all-*trans* configuration, thereby triggering a series of conformational changes in the opsin protein, leading to the formation of the active metarhodopsin II state that initiates signalling in the G-protein cascade of phototransduction. Rapid shut-off of the activated protein is brought about by multiple phosphorylation of metarhodopsin II, followed by arrestin binding. Subsequently, and much more slowly, the covalent bond is hydrolysed, so that the all-*trans* aldehyde is available for reduction by the RDH5 retinol dehydrogenase into the all-*trans* alcohol, vitamin A. The released vitamin A passes out of the photoreceptor outer segment, and is chaperoned, in part by IRBP (interphotoreceptor retinoid-binding protein), to the adjacent retinal pigment epithelium. Here, the vitamin A, which is now chaperoned by CRBP (cellular retinol-binding protein), is esterified by LRAT (lecithin retinol acyl transferase) to all-*trans*-retinyl palmitate, and then isomerohydrolysed by RPE65 (retinal pigment epithelium-associated 65 kDa protein) to the 11-*cis* alcohol, and subsequently oxidized by the RDH12 retinol dehydrogenase to 11-*cis*-retinaldehyde, which is chaperoned by CRALBP (cellular retinal-binding protein). Finally, the 11-*cis* retinaldehyde is transported out of the RPE, across the extracellular space, again chaperoned by IBRP, and back into the photoreceptor outer segment, where it binds to free opsin to re-form the ground state visual pigment. This metabolic pathway for the regeneration of 11-*cis*-retinal is used by both rods and cones, though an additional pathway involving the Müller cells is also used by cones; see Mata *et al*. (2002) and Miyazono *et al*. (2008).

It has been reported that a related retinoid processing system may exist in the tunicate *Ciona*, with the possibility that this organism reflects steps in an evolutionary sequence, from the protostome arrangement, through the *Ciona* larva, and the adult *Ciona*, towards the vertebrate system of retinoid cycling; see, in particular, fig. 5 of Kusakabe *et al*. (2009). However, it needs to be stressed that although a number of apparently homologous proteins have been identified, it has not yet been shown definitively what their functional role is in *Ciona*.

The larva of *Ciona* has a system that appears broadly similar to that found in protostomes. The ciliary photoreceptor cells contain both Ci-opsin3, apparently a homologue of retinochrome which acts as a photoisomerase in protostomes, and also Ci-CRALBP, a homologue of CRALBP which in vertebrates chaperones retinaldehyde within RPE cells. In addition, adjacent non-photoreceptor cells contain not only Ci-opsin3 and Ci-CRALBP, but also Ci-BCO, apparently a homologue of the vertebrate BCO (β-carotene 15,15′-monooxygenase) that cleaves β-carotene into vitamin A. Hence, it is entirely plausible that the *Ciona* larva generates 11-*cis*-retinal solely via a photoisomerase system comparable to that in protostomes, though using a chaperone protein more closely related to the vertebrate version.

The adult *Ciona* has photoreceptors in a cerebral ganglion (amongst several locations). Here, the photoreceptors appear not to contain either a retinoid chaperone protein or a photoisomerase, as is the case with vertebrate photoreceptors. On the other hand, the adjacent non-photoreceptor cells contain (in addition to the larval complement of Ci-BCO, Ci-CRALBP and Ci-opsin3) a presumed homologue of vertebrate RPE65, termed Ci-RPE65 (Kusakabe *et al*. 2009). Thus, although there is as yet no functional evidence, it is entirely plausible that the adult *Ciona* generates 11-*cis*-retinal using a dark isomerization pathway (via Ci-RPE65) as well as possibly using photoisomerization (via Ci-opsin3).

### (b) Ancient evolutionary pressures under low light levels

Extant cephalochordates and tunicates (such as *Branchiostoma* and *Ciona*) typically inhabit relatively shallow water, where the daytime light intensity is high. As early chordates began to colonize deeper waters, where light intensities were much lower, two properties may have been advantageous: an increased sensitivity to light, and an ability to synthesize 11-*cis*-retinal in darkness.

One way of increasing sensitivity at low light intensities is to capture more incident photons, by using more photopigment. This strategy was adopted very early in the evolution of photoreceptors, through the great elaboration of their pigment-containing membrane, in the microvilli of rhabdomeric photoreceptors and in the precursors of the sacs/discs of vertebrate photoreceptors. For an animal coping with very low light levels, an extension of this strategy may simply have been to expand the size of the photoreceptive organs, by increasing the number of photoreceptors and thereby increasing the number of photons absorbed. In early chordates, any expansion of this kind was likely to have been occurring in parallel with the development of a protective cranium (that would shield downwelling light to some extent), and so it is reasonable to think that it would have been advantageous if an outward, lateral, ballooning of the simple paired ocelli (e.g. ‘frontal eyes’) had occurred; this would have provided an enlarged mass of light-sensitive cells, unshielded by the skull (Lamb *et al*. 2007, 2008). In addition to providing improved sensitivity, such a bilateral development of light-sensitive organs would have made possible the provision of an optical sense of body orientation relative to the downwelling light (i.e. a sense of roll, or rotation about the long body axis), through relatively simple neural processing.

A further increase in light sensitivity could have been achieved by shifting the pigmented cells (which absorb incident light) out of the light-absorbing epithelium. If photoreceptor cells could be made to occupy the entire cross-sectional area of the epithelium, thereby increasing the number of photoreceptors per unit area, then more of the incident light would be absorbed. However, because of the need to resynthesize (rather than photo-regenerate) 11-*cis*-retinal for the ciliary opsin, it would have been necessary to have retained the chemical machinery of dark isomerization somewhere nearby. The combination of these two advantages (photoreceptors making up the entire cross-section, yet the chemistry of dark isomerization being nearby) could have readily been achieved by an invagination of the ballooning light-sensitive region, and with a ‘division of labour’ (cf. Arendt *et al*. 2009), whereby the processing of retinoid occurred only in the outer layer, while photoreception and neural processing occurred in the inner layer.

Thus, there is a coherent rationale to explain why the invagination of the ballooning eye vesicle that occurs in the developing vertebrate embryo would have represented an advantageous development in the evolution of the light-sensitive organs of early chordates. It is important to note that, in this rationalization, no case has yet been made for the development of, or the advantage of, directional (i.e. imaging) vision. The argument has been made solely in terms of coping with the lower light levels encountered in deeper waters. In other words, this proposal would fit with the notion that this particular line of evolving chordates faced great pressure to cope with low light levels prior to facing pressure for spatial sensing of illumination (imaging vision).

The arrangement described above corresponds closely to that found in the ‘eyes’ of extant hagfish. The hagfish is a basal craniate, living at great depths in the ocean, and possessing lateral photosensitive organs that have the invaginated eyecup arrangement of vertebrates, though with no signs of optical imaging or of any other developments associated with spatial vision. For detailed discussion of the hagfish eye, see Holmberg (1977); Locket & Jorgensen (1998); and Lamb *et al*. (2007).

If the reasoning above is valid, then why did similar developments occur only in chordates and not in protostomes? The primary factor would seem to have been the inability of chordate ciliary opsins to undergo photoreversal to the ground state, and hence their need for an exogenous source of 11-*cis*-retinal. This necessitates either a separate photoisomerase (as in some protostomes) or a dark isomerase. For animals living near the surface, a separate photoisomerase is sufficient, and appears to be the solution adopted in the tunicate larva. But for animals inhabiting greater depths, there is a requirement for an isomerase that functions in the dark. The use of such an isomerase appears to be one of the solutions adopted by the adult tunicate, which is a sessile animal adhering to rocks on the sea-floor. Once a dark isomerase (i.e. an RPE65 precursor) had evolved in the cells adjacent to the photoreceptors, then ciliary photoreceptors with their *c*-opsins would have held a distinct advantage over rhabdomeric photoreceptors with their *r*-opsins, in the dimmer light conditions encountered in deep water. With very little light present at depth, it would be almost impossible to create the 11-*cis*-retinal needed to begin the process of phototransduction in a rhabdomeric photoreceptor. Instead, what is needed under continual low light levels is not an opsin that is bistable, but instead one that readily releases its all-*trans* chromophore, and is therefore able to employ exogenous 11-*cis*-retinal synthesized by a dark isomerase.

## 4. Origin of vertebrate retinal cells and circuitry

It has been known since the 1980s that retinal progenitor cells appear to be unrestricted in cell fate, in that a single dividing progenitor cell can produce clones comprising any combination of retinal cell types (Turner & Cepko 1987; Holt *et al*. 1988; Wetts & Fraser 1988). Subsequently, Arendt (2003) demonstrated a remarkable homology between vertebrate retinal ganglion cells and the rhabdomeric photoreceptors of protostomes, in terms of transcription factors and expressed proteins (including melanopsin). He proposed that retinal ganglion cells, amacrine cells and horizontal cells are all ‘sister cells’ of rhabdomeric photoreceptors; i.e. that these three classes of vertebrate retinal neurons and invertebrate rhabdomeric photoreceptors are all derived from a common ancestral cell type in a bilateral ancestor. And in the outer retina, the close similarities of vertebrate retinal bipolar cells and photoreceptors led Lamb *et al*. (2007) to propose that bipolar cells are derived from ciliary photoreceptors.

Recently, Arendt (2008) has extended these ideas into a model for the evolution of cell types, using the powerful information that can be obtained from ‘molecular fingerprinting’ of different cell classes, and he has set out several principles for the evolution of cell types. He provides evidence that early metazoans possessed few cell types, but that these cell types were typically multi-functional and expressed numerous genes, and that in the course of evolution, cells tended to diversify by segregation of function. Thus, two descendant (sister) cell types would tend to have the original functions of the parent cell type divided between them, in a complementary manner, so that they would each become more specialized; in addition, each might gain new functions. He further proposed that functionally divergent sister cell types might tend to migrate apart spatially, but that in doing so they would tend to retain contact with each, as exemplified by neural contacts between distant cells in the nervous system.

Based on these ideas, it is possible to set out a hypothetical account of the evolution of cell types and cell connections in the vertebrate retina.

Prior to the separation of protostomes and deuterostomes, an ancestral multi-functional photoreceptor cell type had diversified into two sister cell types, from which all rhabdomeric and ciliary photoreceptors have subsequently evolved. At an early stage in the evolution of chordates, ciliary and rhabdomeric photoreceptors are assumed to have existed in proximity to each other, in the primordial diencephalon (though reports of close proximity in extant chordates are lacking). Both classes of photoreceptor would have made synaptic contact with effector cells, which were possibly neurosecretory cells in the adjacent primordial hypothalamus. The rhabdomeric photoreceptors depolarized to light, and perhaps mediated ‘light-on’ responses, while the ciliary photoreceptors depolarized when light was extinguished and perhaps mediated ‘light-off’ responses.

When early chordates of this kind moved to greater depths in the sea, where light levels were much lower, the rhabdomeric photoreceptors became less capable of signalling light, through lack of the long-wavelength light needed for conversion of metarhodopsin to the ground state pigment (see §3*b*). Hence, it may have become advantageous for the ciliary photoreceptors to make synaptic contact onto the rhabdomeric photoreceptors, and to use their central axonal projections. These modified rhabdomeric photoreceptors would then have served as retinal output neurons (retinal ganglion cells), and for most such neurons there may have been little advantage in retaining their light-transducing function. Why there may have been advantage in the ciliary photoreceptors signalling solely via the (former) rhabdomeric cells, and hence losing any other axonal projections of their own, is not entirely clear; however, if the rhabdomeric axonal pathway was capable of conveying the required information, then a duplicate set of axonal projections may have had negligible benefit but considerable cost.

Diversification of these output neurons (modified from rhabdomeric photoreceptors) may have led to additional cell types, amacrine cells and horizontal cells, which share many of the transcription factors used by ganglion cells (Arendt 2003). Amacrine cells are morphologically and functionally very similar to ganglion cells but do not have axons, and mediate lateral (and other) interactions in the inner retina. Horizontal cells spread tangentially across the retina, and are coupled in a syncitium by gap junctions; they contact photoreceptors, and mediate lateral interactions in the outer retina.

In the ancestral chordate's photoreceptive epithelium, there may have been only a single class of ciliary photoreceptor. But that ancestral ciliary photoreceptor appears to have diversified a number of times. One of its first diversifications may have been the formation of the retinal bipolar cell, through the loss of its light-sensitive outer segment. In the vertebrate retina, bipolar cells share a large repertoire of effector proteins with cone and rod photoreceptors, along with a similar bipolar morphology and radial organization. During development, and in many cases in the adult retina, they even retain the 9 + 0 primary cilium and a structure homologous to the inner segment known as a Landolt club (e.g. Hendrickson 1966; Quesada & Genis-Galvez 1985). In addition, the output synapse of the bipolar cell employs a specialized ‘ribbon synapse’ that is found elsewhere only in photoreceptors and hair cells. What bipolar cells have either retained from their ancestors (or else gained), that extant vertebrate photoreceptors have presumably lost, are glutamatergic synaptic receptors at their distal (dendritic) terminals. At the gene level, it has been proposed that this diversification involved the selective retention only in bipolar cells of two homeobox genes, *Chx10* and *Vsx* (Arendt 2008), though it seems possible that there may additionally have been loss of other components in bipolar cells.

A second set of diversifications occurred, though it is not clear whether these began before or after the advent of bipolar cells: this was the diversification of classes of ciliary opsin and ciliary photoreceptor. It seems likely that this set of diversifications resulted from the two stages of whole genome duplication that occurred somewhere around the base of the vertebrate radiation (see, for example, Nordström *et al*. 2004; Larhammar *et al*. 2009). Furthermore, it is plausible to think that these events were of particular advantage to those chordates returning to shallow waters, from among ancestors that had evolved retinal ganglion cells under the survival pressures operating at sustained low light levels. For animals near the surface there would have been advantage in using the broad range of wavelengths available, and also advantage in comparing wavelength composition; i.e. in using colour information. In any case, an early vertebrate, the last common ancestor of lampreys and jawed vertebrates, possessed five classes of cone opsin (Collin *et al*. 2003). In view of the fact that in some extant lampreys as well as in many extant jawed vertebrates, these five classes of opsin are differentially expressed in five morphologically separate classes of ciliary photoreceptor (Davies *et al*. 2007; Collin *et al*. 2009), it seems very likely that the five classes of cone-like photoreceptors had already been established in that ancestor.

Hence, it seems firmly established that, prior to the divergence of jawless and jawed vertebrates, the proto-vertebrate retina was already fundamentally in the form of the present photopic (cone) retina. It possessed five classes of cone-like ciliary photoreceptor, retinal bipolar cells of ON and OFF divisions, horizontal cells, amacrine cells and ganglion cells, and it seems likely that it performed signal-processing operations very similar to those of the modern lamprey and jawed-vertebrate retinas. The advantages provided by these developments were numerous, and included: spatial summation of signals, spatial contrast signalling and probably colour processing; most importantly, though, by this stage the retina could provide the brain with the information required for spatial vision.

The acquisition of these characteristics occurred with remarkable rapidity, within the time boundaries set by the divergence from the future gnathostome line of (i) tunicates, perhaps 550 Ma and (ii) Petromyzoniformes, at the latest 500 Ma. If it becomes possible to establish with certainty the phylogenetic position of hagfish, then it may be possible to delineate sub-divisions in the timing of the acquisition of these characteristics more clearly (see Lamb *et al*. 2007).

## 5. Origin of vertebrate scotopic vision

At the stage of evolution of the proto-vertebrate retina described in the preceding section, when the ancestors of lampreys and jawed vertebrates diverged, it seems almost certain that the scotopic (rod-based) capability of modern vertebrates had not yet evolved; instead, the retina was based solely on the pathways of the modern photopic (cone-based) sub-division. The modern duplex rod/cone retina was established by the time of the last common ancestor that we share with cartilaginous fish, because (for example) the dogfish retina contains rods and rod bipolar cells whose function is essentially indistinguishable from that of our own (Ashmore & Falk 1980). Next, I consider separately a number of specializations: of the rod photoreceptor cell, of the rhodopsin molecule, of the other components of transduction, and of the subsequent retinal circuitry.

### (a) Specializations of the rod photoreceptor cell

In order to describe the specializations that characterize rods, it is first necessary to define what is meant by a rod photoreceptor. Although the distinction is clear in the mammalian retina, there are cases where the situation is blurred, as is especially the case in the lamprey retina. Here, a rod is defined functionally, as a vertebrate ciliary photoreceptor that is reliably able to detect the arrival of individual photons of light. In contrast, a cone is defined here as a vertebrate ciliary photoreceptor that has a rapid response to light, and is able to light-adapt over an enormously wide range of operating intensities, so that in practice it is never saturated by the application of steady illumination, no matter how bright.

Rod photoreceptors typically employ a ‘rod’ opsin (rhodopsin), and typically have a cylindrically shaped outer segment, in which the great majority of plasma membrane out-foldings have become sealed-off as free-floating ‘discs’, and only a relatively small number of the most basal membrane foldings remain as patent ‘sacs’. In cones, the shape of the outer segment is typically conical in non-mammalian species, though in mammals it is usually cylindrical. The electrical response of a rod to light is invariably slower and more sensitive than the response of a cone in the same retina. But the rod is only capable of functioning at very low light intensities, and it saturates (i.e. all its outer segment channels are closed) at intensities corresponding to twilight levels. Furthermore, a rod recovers from intense ‘bleaching’ light exposures far more slowly than does a cone. Indeed, upon extinction of steady illumination bleaching 90 per cent of the visual pigment, the time for complete recovery of circulating current in mammalian photoreceptors is around 20 min in a rod (Thomas & Lamb 1999), but only 20 ms in a cone (Kenkre *et al*. 2005). This difference, a factor of 60 000-fold, is the greatest known difference in properties between mammalian rod and cone photoreceptors. While we do not fully understand the basis for the difference, it is related to the fact that the very sensitive rods are much more susceptible to the presence of photoproducts (such as opsin), and that the final ‘elimination’ of opsin is determined by the delivery and binding of 11-*cis*-retinal (Lamb & Pugh 2004).

It is remarkable that we still do not understand the functional significance of the major morphological difference between rods and cones: the possession by rods of sealed-off discs. It seems highly likely that the rod's ability to reliably detect individual photons is somehow associated with the presence of free-floating discs, but at present we can only speculate on the advantage conveyed by discs. With the disc interior sealed off from the extracellular medium, the electrical capacitance of the outer segment of a rod is much smaller than that of a cone, but it is difficult to see how this could contribute to single-photon detection, and furthermore the larger capacitance of cones is the opposite of what is required for rapid responses. One possibility is that the disc interior has a different ionic composition, or a different pH, from the extracellular medium; however, this proposition is difficult to evaluate because we know neither the ionic composition of the extracellular medium in the narrow gap between the photoreceptor outer segments and the retinal pigment epithelium, nor that within the discs.

Despite the lack of a convincing explanation for the mechanism by which discs provide an advantage, it seems likely that the organization into pinched-off discs is crucial to the rod's operation. Firstly, there are no examples of vertebrate photoreceptors with patent sacs (i.e. cones) that are able to reliably detect individual photons. Secondly, there is a well-established case of a ‘morphological rod’ containing a ‘cone pigment’, which exhibits response properties essentially indistinguishable from any other rod.

Thus, the amphibian ‘green rod’ expresses an *SWS2* opsin (Hisatomi *et al*. 1999), identical to that expressed in the blue-sensitive cones of the same species (Ma *et al*. 2001; Takahashi *et al*. 2001). This rod is so-named because of its pale green colour, which stems from the absorption of its pigment in the blue region of the spectrum. The rate of thermal isomerization in the green rods of the toad is only a factor of 5 higher than that in the rhodopsin-containing red rods of the same species (Matthews 1984), at a very low value of approximately 5 × 10^−11^ s^−1^ per pigment molecule. This is orders of magnitude lower than the rate of pigment activation calculated for long-wave-sensitive (LWS) cones from noise measurement (Lamb & Simon 1977; Rieke & Baylor 2000). It has not, however, yet been possible to establish the rate of pigment activation in cones containing a short-wave sensitive pigment (SWS1, SWS2 or Rh2), because the noise is dominated by downstream events in the transduction cascade (Rieke & Baylor 2000).

A final specialization of rod morphology that deserves mention involves the nuclear architecture in rods of nocturnal mammals that has been reported very recently by Solovei *et al*. (2009). In nocturnal species, the arrangement of chromatin in the nucleus is inverted from normal, altering the refractile properties and causing the nuclei (which are arranged in columns) to act as tiny lenses. The authors argue that this causes incident light to be channelled into the outer segments, though it is difficult to see how this could be advantageous at the single-photon level. On the other hand, if this arrangement caused a reduction in the attenuation or the scattering of incident light, then it would indeed be of advantage to the scotopic system.

### (b) Specializations of the rhodopsin visual pigment molecule

As was established by Okano *et al*. (1992), and as is illustrated in figure 2*b*, rod opsin arose after four classes of cone pigment had already evolved (see also Shichida & Matsuyama 2009). Within the group of vertebrate retinal ciliary opsins, the first split was into a LWS opsin (in which the primordial E181 counterion was replaced by a chloride-binding site) and a short-wavelength-sensitive opsin. The latter subsequently split again to form SWS1 opsin and a mid-wavelength-sensitive opsin, which in turn split into the SWS2 opsin and another opsin, which again diverged into the Rh2 (cone) opsin and Rh1 (rod) opsin.

It is often thought that a characterizing feature of rod opsin in comparison with cone opsins is its enormously greater stability against thermal activation (see §5*a*). However, this result has only been established in comparison with the LWS cone opsin, and it is entirely possible that each of the other cone opsins (SWS1, SWS2 and Rh2) exhibit thermal stability comparable to rod opsin. If this proves to be the case, then it would suggest that the replacement of E181 with a chloride-binding site could be a prime suspect for the difference in stability. Such a result would further suggest that the ability of the photoreceptor cell to take full advantage of the great stability of the visual pigment molecule, and thereby become a single-photon detector, did not evolve until long after the molecule had become sufficiently thermally stable.

In order to determine the sites that are important for distinguishing cone and rod opsins, one can compare sequences across opsin families and look for differences, and then apply site-directed mutagenesis. Two such important sites were identified by Shichida's group: site 122, which is Glu in rods but not cones (Imai *et al*. 1997), and site 189, which is Pro in cones and Ile in rods (Kuwayama *et al*. 2002; figure 2*c*). Site-directed mutagenesis showed that the combination of effects of cone versus rod residues at these two sites led to a large (approx. 100-fold) difference in the decay rate of metarhodopsin II; the rod combination E122/I189 gave a far slower rate of metarhodopsin II decay than did the cone combination Q122/P189 (Kuwayama *et al*. 2005; reviewed in Imai *et al*. 2005 and Shichida & Matsuyama 2009). Thus, these two sites are able to account for the difference in the rate of hydrolysis of the chromophore Schiff-base bond between rods and cones. Furthermore, these studies additionally showed that the two sites have a major effect on the rate at which opsin recombines with 11-*cis*-retinal, further accounting for the differences between the properties of rod and cone opsin.

Carleton *et al*. (2005) employed evolutionary trace analysis of a set of 188 vertebrate retinal ciliary opsins to investigate the functionally important sites in each of the five classes of pigment. Although they could identify ‘signature sites’ in each of the cone opsins, where the amino acid residue was unique to the class of pigment, they were surprised to find that rod opsin had no such signature sites. In other words, although around half of the rod opsin's sites were unanimous across all the species examined, the amino acid at any one of those sites could also be found in at least one of the cone opsin classes. Thus, in examining a larger set of rod opsins, they did not see the strict conservation of E122 and I189 found by Shichida's group; they reported that site 189 of the rod opsin was either Ile or Gln in two of the species examined, while site 122 was Ile in only about 50 per cent of cases, and Val in the remainder. Hence, they reported that it was not possible to characterize rod opsins as distinct from cone opsins on the basis of individual sites.

Accordingly, it is possible to make a minor change to the interpretation of both groups as follows. The sites 122 and 189 can be considered as characteristic for rods when (with only a few exceptions) site 122 is Glu and when site 189 is either Ile or Val (which have very similar biochemical properties).

For the future, it will be of great interest to elucidate which sites determine the great stability of the rod opsin molecule against thermal isomerization, and which sites contribute to other distinct properties of intact rods and cones recorded electrophysiologically.

### (c) Specializations of other phototransduction proteins in the rod

The phototransduction cascade in cones and rods is fundamentally the same. The visual pigment is a GPCR which, when isomerized by light, activates multiple copies of a G-protein, transducin. This binds to and activates a phosphodiesterase (PDE), leading to reduced levels of cyclic GMP in the cytoplasm, and thereby to closure of cyclic nucleotide-gated channels in the plasma membrane. The activated visual pigment is shut-off rapidly by the combination of multiple phosphorylation by a G-protein receptor kinase and binding of arrestin; the final, but much more slower, shut-off is brought about by hydrolysis of the all-*trans* chromophore followed by recombination of opsin with 11-*cis*-retinal. Shut-off of the G-protein is greatly accelerated, after it has bound the PDE, by the further binding of an RGS (regulator of G-protein signalling) protein. Recovery of cGMP levels, and light-adaptation, are assisted by the drop in cytoplasmic calcium level that is brought about by closure of outer segment channels. The reduced calcium level leads both to activation of guanylyl cyclase-activating proteins, which stimulate guanylyl cyclase to synthesize more cGMP, and also through an action on a protein that regulates the binding affinity of the outer segment ion channels for cGMP. All these statements apply both to cones and rods.

Many of the proteins in rods and cones are identical, though there are also many cases where different isoforms are employed in the two classes. For example, the G-protein α subunit is GNAT1 in rods, but GNAT2 in cones, while the β subunit is GNB1 in rods but GNB3 in cones. Of considerable interest to the evolution of retinal bipolar cells is the fact that many of the same protein components are used, as are found in either cones or rods. A detailed account of the differences in all the components of transduction is beyond the scope of this paper, as is an account of the phylogenies of the entire complement of transduction cascade components. Excellent reviews of the molecular evolution of these components have been provided by Hisatomi & Tokunaga (2002), and by Larhammar *et al*. (2009); the latter paper emphasizes the role of genome duplications in the evolution of the components.

### (d) Contributions to cone/rod differences

Overall, we know a great deal about the minutiae of the substantial molecular and morphological differences between cones and rods. Although we cannot yet provide a detailed account of how these differences contribute to the functional differences between cones and rods, it is possible to make the following generalizations.

In cones, a rapid response is crucial, and this is presumably brought about by the combination of isoforms of transduction cascade components (including opsin) that each contribute to rapid termination of light-induced activity. For example, the shut-off of activated photopigment in mammalian cones is 20-fold faster than that in mammalian rods (approx. 4 ms; cf. 70 ms) and the shut-off of activated transducin/PDE is also 20-fold faster (approx. 10 ms; cf. 200 ms). It has been shown that this combined 400-fold difference can account for the ability of mammalian cones to avoid saturation, no matter how intense the steady light becomes (Lamb & Pugh 2006).

Rods, on the other hand, need to integrate the effects of each photoisomerization, and they need an exceptionally stable photopigment (i.e. one that has an extremely low rate of thermal activation). The greater response summation appears to be brought about by a combination of isoforms of the cascade components (including opsin) that provide slower termination of light-induced activity. The greater thermal stability is a property of the photopigment molecule, and may in fact be present in all the ‘shorter-wave’ pigment classes (SWS1, SWS2, Rh2 and Rh1); i.e. it may only be the LWS pigment, with its chloride-binding site replacing E181, that exhibits substantially poorer thermal stability.

### (e) Specializations of the rod circuitry

The existence of somewhat unusual retinal circuitry in the rod pathway of mammals was first described by Kolb & Famiglietti (1974), and the details of this circuitry were expanded upon by Strettoi *et al*. (1990); Vaney *et al*. (1991) and Wässle *et al*. (1991). Coincidentally, it was in 1992, the year of the discovery that rod opsins had evolved from cone opsins, that Strettoi *et al*. (1992) set out the concept that the mammalian scotopic retinal pathway ‘piggybacks’ on the conventionally understood cone pathway.

In functional terms, the advantages of this arrangement are that the duplex cone/rod system does not require two separate pathways of neural processing in the retina together with two sets of nerve fibres to the brain. Instead, the rod signals are admitted into the pre-existing and sophisticated cone neural-processing circuitry where they can be seamlessly integrated; perceptually, this means that it is almost impossible for one to distinguish whether one's scotopic or photopic system is being used.

**Figure 3.**
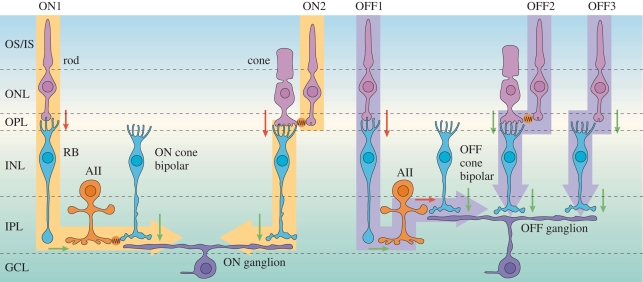
Schematic wiring diagram of the mammalian retina, emphasizing the rod (scotopic) pathways. Left, ON pathways. Right, OFF pathways. Cone circuitry is indicated using just two cone photoreceptors; that in the left half is shown connecting via an ON cone bipolar cell to an ON ganglion cell; that in the right half is shown connecting via an OFF cone bipolar cell to an OFF ganglion cell. The rod pathways are described in the text. Adapted with permission from Wässle (2004).

The manner in which the rod signals are injected into the mammalian cone system may appear complicated (figure 3), but it cleverly avoids introducing major compromises, especially in relation to the possible contribution of additional noise. First of all, from mesopic (twilight) light levels down to high scotopic (moonlight) levels, the rod signals pass via gap junctions onto cones (denoted ON2 and OFF2 in figure 3), thereby employing the photopic pathway in its entirety. But at very low scotopic (starlight) levels, a separate dedicated system comes into operation, using the rod bipolar cell and the AII amacrine cell.

For the ON pathway, illustrated on the left side of figure 3, the photopic pathways uses a sign-inverting synapse (red arrow) from cone to cone ON bipolar cell (via a metabotropic glutamate receptor mechanism), followed by a sign-preserving synapse (green) from cone ON bipolar cell to ON ganglion cell. Thus, at low light levels, when the cones are contributing little in the way of signals, the ON bipolar cell is hyperpolarized and the synapse from ON bipolar cell to ganglion cell is quiescent. At low scotopic levels (indicated ON1), rod activity is signalled via a sign-inverting synapse onto the rod bipolar cell, and thence via a sign-preserving synapse to the AII amacrine cell, so that dim scotopic illumination causes depolarization of the AII amacrine cells (i.e. the same polarity as any light-stimulated activity in the cone ON bipolar cells). The scotopic signals are then coupled into the cone pathway, with no additional noise contribution, via gap junction electrical connections from the AII amacrine cells onto the cone ON bipolar cells (indicated in orange). In bright light, when the rods are saturated, and the rod bipolar cells and AII amacrine cells are strongly depolarized, it is possible that the gap junctions close, to prevent the intrusion of unwanted signals. The alternative possibility, of connecting rod bipolar cells directly onto ON ganglion cells, would not allow this kind of regulation, and would suffer from injecting large levels of noise into the photopic system when the rods were saturated.

For the OFF pathway, illustrated on the right of figure 3, the photopic pathways uses a sign-preserving synapse (green arrow) from cone to OFF bipolar cell (via an ionotropic glutamate receptor mechanism), followed by a sign-preserving synapse (green) from cone OFF bipolar cell to OFF ganglion cell. As a result, in the quiescent dark state, the cone ON bipolar cell is depolarized and is continually releasing synaptic transmitters (glutamate) onto the OFF ganglion cell at a high rate. How, then, could activity from the scotopic system be injected into the cone OFF pathway, in the presence of this continual synaptic input? The answer that has been adopted is to employ a sign-inverting synaptic input from the AII amacrine cell onto the pre-synaptic terminal of the cone ON bipolar cell (indicated OFF1). In this way, dim scotopic light leads to hyperpolarization of the cone ON bipolar cell's synaptic terminal, thereby suppressing its ongoing high rate of release of synaptic transmitter, just as occurs for signals that come from the cones.

In other words, evolution found a means by which to superimpose the single-photon detection capabilities of the more recently evolved rods onto a pre-existing cone neural processing pathway, in a seamless manner, with minimal additional wiring, and in a way that introduces minimal additional noise and that minimally perturbs the sophisticated signal-processing functions of the cone circuitry.

It would be very interesting to know whether similar AII amacrine cell circuitry is employed in the retinae of vertebrates other than mammals, but so far a definitive answer to this question does not appear to have been established. In non-mammalian retinae, it is clear that scotopic signals are processed via bipolar cells that are highly sensitive and that display responses very similar to those of mammalian rod bipolar cells (e.g. Ashmore & Falk 1980). However, the circuitry by which rod and cone signals are combined is not entirely clear. One possibility is that rod and cone signals are merged solely at the level of the outer plexiform layer, via gap junctions between rods and cones, and possibly via synaptic input from both rods and cones onto a given bipolar cell. In this case the bipolar cells would be of the mixed rod-cone type, and thereafter the signals would be processed by the conventional cone pathway of bipolar cell to ganglion cell. Alternatively, it is possible that circuitry analogous to that of the AII amacrine cell exists in the inner retina. Until the details of the scotopic circuitry of non-mammalian vertebrates species have been elucidated, it may not be possible to ascertain the extent to which the early vertebrate retina employed the outer versus the inner plexiform layer to inject its rod-derived signals into the cone pathway. But in either case it is clear that, as a minimum, the output (ganglion cell) stage of the pre-existing cone system was used.

## 6. Summary: a scenario for the evolution of vertebrate retinal photoreception

Combining the information set out above, it is possible to present the following as a likely scenario for the sequence of events by which vertebrate retinal photoreception arose.

Prior to the divergence of protostomes and deuterostomes, a primordial photoreceptor with an animal opsin and a G-protein signalling cascade had duplicated, so that bilateral animals possessed two closely related variants: (i) a rhabdomeric-style photoreceptor with an *r*-opsin and a cascade that probably used a G_q_-based G-protein and (ii) a ciliary-style photoreceptor with a *c*-opsin and a cascade that probably used a forerunner of transducin coupling to a PDE.

In chordate evolution, the *c*-opsin gained an alternative counterion site at E113, and it lost the capacity to undergo photoreversal to the ground state. Under this pressure, a dark re-isomerization pathway evolved in chordates, in which a forerunner of RPE65 most likely evolved from BCO. In the battle to occupy deeper environments, with perpetually low light levels, these chordates had an advantage over organisms using *r*-opsins, in that their photoreceptors could still function, because of the dark retinoid isomerase activity. In response to the lowered light levels, their bilateral photosensitive areas in the proto-diencephalon expanded laterally to absorb more of the incident photons. With their rhabdomeric photoreceptors inoperative (due to their inability to re-isomerize photopigment in the absence of light), those connections to the central nervous system were taken advantage of through the development of synaptic input from the ciliary photoreceptors.

In parallel, the tasks of photoreception and dark re-isomerization were relegated to neighbouring regions of cells, so that the light-absorbing epithelium solely contained photoreceptors and thereby absorbed more of the incident light. This had to be achieved while keeping the two sets of cells in close proximity, and this occurred through an invagination of the developing optic vesicle, bringing the proto-retina into apposition with the proto-RPE. At this stage of chordate evolution (possibly similar to the situation in extant hagfish), the bilateral retinae had a primarily circadian function; i.e. they were not involved in imaging vision.

Subsequently, possibly in association with two stages of whole genome duplication, multiple classes of *c*-opsin evolved, along with multiple classes of ciliary (cone) photoreceptors. A major evolutionary advance occurred when one class of ciliary photoreceptor became specialized to receive synaptic input from other ciliary photoreceptors, thereby giving rise to the cell class of retinal bipolar cells. The great increase in retinal processing power that this enabled then made it a relatively simple matter for the retina to compute spatial contrasts. In this situation, the development of any kind of lens-like properties as a result of thickening of the overlying surface of the animal could readily have led to simple spatial visual information being conveyed to the brain. Subsequent perfection of the optical properties could have occurred with great rapidity. At this stage of vertebrate evolution, the lateral eyes may have been fundamentally similar to those of extant lampreys.

For these animals, their spatial vision would have been effective under illumination from daylight levels down to twilight levels. Such animals, whose photoreceptors developed the ability to make use of the enormous thermal stability of the shorter-wave-sensitive *c*-opsins, and thereby reduce the receptor noise levels to the point where it became possible to detect single photons, would have had a great advantage at night and in deep water. Rod photoreceptors with the requisite properties evolved, and the neural wiring of the retina evolved in such a way that their signals were able to piggyback onto the existing cone system.

The above sequence of events is at least a plausible description of the way that our retina evolved. Consideration of the ideas presented here leads to the suggestion of a variety of experiments that could be conducted (some of which were set out in Lamb *et al*. 2007), in order to test the validity of the proposed scenario.

## References

[RSTB20090102C1] Arendt D. 2003 Evolution of eyes and photoreceptor cell types. Int. J. Dev. Biol. 47, 563–571 14756332

[RSTB20090102C2] Arendt D. 2008 The evolution of cell types in animals: emerging principles from molecular studies. Nat. Rev. Genet. 9, 868–882 (doi:10.1038/nrg2416) 18927580 10.1038/nrg2416

[RSTB20090102C3] Arendt D. Wittbrodt J. 2001 Reconstructing the eyes of Urbilateria. Phil. Trans. R. Soc. Lond. B 356, 1545–1563 (doi:10.1098/rstb.2001.0971) 11604122 10.1098/rstb.2001.0971 PMC1088535

[RSTB20090102C4] Arendt D. Tessmar-Raible K. Snyman H. Dorresteijn A. W. Wittbrodt J. 2004 Ciliary photoreceptors with a vertebrate-type opsin in an invertebrate brain. Science 306, 869–871 (doi:10.1126/science.1099955) 15514158 10.1126/science.1099955

[RSTB20090102C5] Arendt D. Hausen H. Purschke G. 2009 The ‘division of labour’ model of eye evolution. Phil. Trans. R. Soc. B 364, 2809–2817 (doi:10.1098/rstb.2009.0104) 19720646 10.1098/rstb.2009.0104 PMC2781865

[RSTB20090102C6] Arnis S. Hofmann K. P. 1995 Photoregeneration of bovine rhodopsin from its signaling state. Biochemistry 34, 9333–9340 (doi:10.1021/bi00029a008) 7626602 10.1021/bi00029a008

[RSTB20090102C7] Arnis S. Fahmy K. Hofmann K. P. Sakmar T. P. 1994 A conserved carboxylic acid group mediates light-dependent proton uptake and signaling by rhodopsin. J. Biol. Chem. 269, 23879–23881 7929034

[RSTB20090102C8] Ashmore J. F. Falk G. 1980 Responses of rod bipolar cells in the dark-adapted retina of the dogfish, *Scyliorhinus canicula*. J. Physiol. 300, 115–150 7381782 10.1113/jphysiol.1980.sp013155 PMC1279348

[RSTB20090102C9] Barnes S. N. 1971 Fine structure of the photoreceptor and cerebral ganglion of the tadpole larva of *Amaroucium constellatum* (Verril) (subphylum: Urochordata; class: Ascidiacea). Z. Zellforsch. Mikrosk. Anat. 117, 1–16 (doi:10.1007/BF00331097) 5559422 10.1007/BF00331097

[RSTB20090102C10] Carleton K. L. Spady T. C. Cote R. H. 2005 Rod and cone opsin families differ in spectral tuning domains but not signal transducing domains as judged by saturated evolutionary trace analysis. J. Mol. Evol. 61, 75–89 (doi:10.1007/s00239-004-0289-z) 15988624 10.1007/s00239-004-0289-z

[RSTB20090102C11] Collin S. P. Knight M. A. Davies W. L. Potter I. C. Hunt D. M. Trezise A. E. O. 2003 Ancient colour vision: multiple opsin genes in the ancestral vertebrates. Curr. Biol. 13, R864–R865 (doi:10.1016/j.cub.2003.10.044) 14614838 10.1016/j.cub.2003.10.044

[RSTB20090102C12] Collin S. P. Davies W. L. Hart N. S. Hunt D. M. 2009 The evolution of early vertebrate photoreceptors. Phil. Trans. R. Soc. B 364, 2925–2940 (doi:10.1098/rstb.2009.0099) 19720654 10.1098/rstb.2009.0099 PMC2781863

[RSTB20090102C13] Davies W. L. Cowing J. A. Carvalho L. S. Potter I. C. Trezise A. E. O. Hunt D. M. Collin S. P. 2007 Functional characterization, tuning, and regulation of visual pigment gene expression in an anadromous lamprey. FASEB J. 21, 2713–2724 (doi:10.1096/fj.06-8057com) 17463225 10.1096/fj.06-8057com

[RSTB20090102C14] Dilly P. Wolken J. 1973 Studies on the receptors in *Ciona intestinalis*. IV. The ocellus in the adult. Micron 4, 11–29

[RSTB20090102C15] Eakin R. M. 1965 Evolution of photoreceptors. Cold Spr. Harb. Symp. Quant. Biol. 30, 363–370 10.1101/sqb.1965.030.01.036 5219487

[RSTB20090102C16] Eakin R. M. Westfall J. A. 1962 Fine structure of photoreceptors in the hydromedusan, *Polyorchis penicillatus*. Proc. Natl Acad. Sci. USA 48, 826–833 (doi:10.1073/pnas.48.5.826) 16590949 10.1073/pnas.48.5.826 PMC220861

[RSTB20090102C17] Hendrickson A. 1966 Landolt's club in the amphibian retina: a Golgi and electron microscope study. Invest. Ophthalmol. Vis. Sci. 5, 484–496 4162899

[RSTB20090102C18] Hisatomi O. Tokunaga F. 2002 Molecular evolution of proteins involved in vertebrate phototransduction. Comp. Biochem. Physiol. B 133, 509–522 (doi:10.1016/S1096-4959(02)00127-6) 12470815 10.1016/s1096-4959(02)00127-6

[RSTB20090102C19] Hisatomi O. Takahashi Y. Taniguchi Y. Tsukahara Y. Tokunaga F. 1999 Primary structure of a visual pigment in bullfrog green rods. FEBS Lett. 447, 44–48 (doi:10.1016/S0014-5793(99)00209-4) 10218579 10.1016/s0014-5793(99)00209-4

[RSTB20090102C20] Holmberg K. 1977 The cyclostome retina. In Handbook of sensory physiology. *Vol VII/5:* The visual system of vertebrates (ed. Crescitelli F.), pp. 47–66 Berlin, Germany: Springer

[RSTB20090102C21] Holt C. E. Bertsch T. W. Ellis H. M. Harris W. A. 1988 Cellular determination in the Xenopus retina is independent of lineage and birth date. Neuron 1, 15–26 (doi:10.1016/0896-6273(88)90205-X) 3272153 10.1016/0896-6273(88)90205-x

[RSTB20090102C22] Horie T. Sakurai D. Ohtsuki H. Terakita A. Shichida Y. Usukura J. Kusakabe T. Tsuda M. 2008 Pigmented and nonpigmented ocelli in the brain vesicle of the ascidian larva. J. Comp. Neurol. 509, 88–102 (doi:10.1002/cne.21733) 18421706 10.1002/cne.21733

[RSTB20090102C23] Imai H. Kojima D. Oura T. Tachibanaki S. Terakita A. Shichida Y. 1997 Single amino acid residue as a functional determinant of rod and cone visual pigments. Proc. Natl Acad. Sci. USA 94, 2322–2326 (doi:10.1073/pnas.94.6.2322) 9122193 10.1073/pnas.94.6.2322 PMC20086

[RSTB20090102C24] Imai H. Kuwayama S. Onishi A. Morizumi T. Chisakac A. Shichida Y. 2005 Molecular properties of rod and cone visual pigments from purified chicken cone pigments to mouse rhodopsin *in situ*. Photochem. Photobiol. Sci. 4, 667–674 (doi:10.1039/b416731g) 16121275 10.1039/b416731g

[RSTB20090102C25] Jeffery W. R. 2004 Evolution and development of brain sensory organs in molgulid ascidians. Evol. Dev. 6, 170–179 (doi:10.1111/j.1525-142X.2004.04022.x) 15099304 10.1111/j.1525-142X.2004.04022.x

[RSTB20090102C26] Kenkre J. S. Moran N. A. Lamb T. D. Mahroo O. A. R. 2005 Extremely rapid recovery of human cone circulating current at the extinction of bleaching exposures. J. Physiol. 567, 95–112 (doi:10.1113/jphysiol.2005.088468) 15932890 10.1113/jphysiol.2005.088468 PMC1474162

[RSTB20090102C27] Knierim B. Hofmann K. P. Ernst O. P. Hubbell W. L. 2007 Sequence of late molecular events in the activation of rhodopsin. Proc. Natl Acad. Sci. USA 104, 20 290–20 295 (doi:10.1073/pnas.0710393104) 10.1073/pnas.0710393104 PMC2154424 18077356

[RSTB20090102C28] Kolb H. Famiglietti E. V. 1974 Rod and cone pathways in the inner plexiform layer of cat retina. Science 186, 47–49 (doi:10.1126/science.186.4158.47) 4417736 10.1126/science.186.4158.47

[RSTB20090102C29] Koyanagi M. Terakita A. 2008 Gq-coupled rhodopsin subfamily composed of invertebrate visual pigment and melanopsin. Photochem. Photobiol. 84, 1024–1030 (doi:10.1111/j.1751-1097.2008.00369.x) 18513236 10.1111/j.1751-1097.2008.00369.x

[RSTB20090102C30] Koyanagi M. Takano K. Tsukamoto H. Ohtsu K. Tokunaga F. Terakita A. 2008 Jellyfish vision starts with cAMP signaling mediated by opsin-Gs cascade. Proc. Natl Acad. Sci. USA 105, 15 576–15 580 (doi:10.1073/pnas.0806215105) 10.1073/pnas.0806215105 PMC2563118 18832159

[RSTB20090102C31] Kozmik Z. 2008 Assembly of the cnidarian camera-type eye from vertebrate-like components. Proc. Natl Acad. Sci. USA 105, 8989–8993 (doi:10.1073/pnas.0800388105) 18577593 10.1073/pnas.0800388105 PMC2449352

[RSTB20090102C32] Kusakabe T. Kusakabe R. Kawakami I. Satou Y. Satoh N. Tsuda M. 2001 *Ci-opsin1*, a vertebrate-type opsin gene, expressed in the larval ocellus of the ascidian Ciona intestinalis. FEBS Lett. 506, 69–72 (doi:10.1016/S0014-5793(01)02877-0) 11591373 10.1016/s0014-5793(01)02877-0

[RSTB20090102C33] Kusakabe T. G. Takimoto N. Jin M. Tsuda M. 2009 Evolution and the origin of the visual retinoid cycle in vertebrates. Phil. Trans. R. Soc. B 364, 2897–2910 (doi:10.1098/rstb.2009.0043) 19720652 10.1098/rstb.2009.0043 PMC2781855

[RSTB20090102C34] Kuwayama S. Imai H. Hirano T. Terakita A. Shichida Y. 2002 Conserved proline residue at position 189 in cone visual pigments as a determinant of molecular properties different from rhodopsins. Biochemistry 41, 15 245–15 252 (doi:10.1021/bi026444k) 12484762 10.1021/bi026444k

[RSTB20090102C35] Kuwayama S. Imai H. Morizumi T. Shichida Y. 2005 Amino acid residues responsible for the meta-III decay rates in rod and cone visual pigments. Biochemistry 44, 2208–2215 (doi:10.1021/bi047994g) 15697246 10.1021/bi047994g

[RSTB20090102C36] Lacalli T. C. 2004 Sensory systems in amphioxus: a window on the ancestral chordate condition. Brain Behav. Evol. 64, 148–162 (doi:10.1159/000079744) 15353907 10.1159/000079744

[RSTB20090102C37] Lamb T. D. Pugh E. N. Jr 2004 Dark adaptation and the retinoid cycle of vision. Prog. Retinal Eye Res. 23, 307–380 (doi:10.1016/j.preteyeres.2004.03.001) 10.1016/j.preteyeres.2004.03.001 15177205

[RSTB20090102C38] Lamb T. D. Pugh E. N. Jr 2006 Avoidance of saturation in human cones is explained by very rapid inactivation reactions and pigment bleaching. Invest. Ophthalmol. Vis. Sci. 47, E-Abstract 3714 (doi:10.1167/iovs.06-0849)

[RSTB20090102C39] Lamb T. D. Simon E. J. 1977 Analysis of electrical noise in turtle cones. J. Physiol. 272, 435–468 592199 10.1113/jphysiol.1977.sp012053 PMC1353567

[RSTB20090102C40] Lamb T. D. Collin S. P. Pugh E. N. Jr 2007 Evolution of the vertebrate eye: opsins, photoreceptors, retina, and eye-cup. Nat. Rev. Neurosci. 8, 960–975 (doi:10.1038/nrn2283) 18026166 10.1038/nrn2283 PMC3143066

[RSTB20090102C41] Lamb T. D. Pugh E. N. Jr Collin S. P. 2008 The origin of the vertebrate eye. Evol.: Educ. Outreach 8, 415–426

[RSTB20090102C42] Lamb T. D. Arendt D. Collin S. P. 2009 The evolution of phototransduction and eyes. Phil. Trans. R. Soc. B 364, 2791–2793 (doi:10.1098/rstb.2009.0106) 19720644 10.1098/rstb.2009.0106 PMC2781866

[RSTB20090102C43] Larhammar D. Nordström K. Larsson T. A. 2009 Evolution of vertebrate rod and cone phototransduction genes. Phil. Trans. R. Soc. B 364, 2867–2880 (doi:10.1098/rstb.2009.0077) 19720650 10.1098/rstb.2009.0077 PMC2781860

[RSTB20090102C44] Locket N. A. Jorgensen J. M. 1998 The eyes of hagfishes. In The biology of hagfishes, ch. 34 (eds Jorgensen J. M. Lomholt J. P. Weber R. E. Malte H.), pp. 541–546 London, UK: Chapman & Hall

[RSTB20090102C45] Ma J. X. 2001 A visual pigment expressed in both rod and cone photoreceptors. Neuron 32, 451–461 (doi:10.1016/S0896-6273(01)00482-2) 11709156 10.1016/s0896-6273(01)00482-2

[RSTB20090102C46] Martin V. J. 2002 Photoreceptors of cnidarians. Can. J. Zool. 80, 1703–1722 (doi:10.1139/z02-136)

[RSTB20090102C47] Mata N. L. Radu R. A. Clemmons R. S. Travis G. H. 2002 Isomerization and oxidation of vitamin A in cone-dominant retinas: a novel pathway for visual pigment regeneration in daylight. Neuron 36, 69–80 (doi:10.1016/S0896-6273(02)00912-1) 12367507 10.1016/s0896-6273(02)00912-1 PMC2851622

[RSTB20090102C48] Matthews G. 1984 Dark noise in the outer segment membrane current of green rod photoreceptors from toad retina. J. Physiol. 349, 607–618 6429322 10.1113/jphysiol.1984.sp015176 PMC1199357

[RSTB20090102C49] Miyazono S. Shimauchi-Matsukawa Y. Tachibanaki S. Kawamura S. 2008 Highly efficient retinal metabolism in cones. Proc. Natl Acad. Sci. USA 105, 16 051–16 056 (doi:10.1073/pnas.0806593105) 10.1073/pnas.0806593105 PMC2572916 18836074

[RSTB20090102C50] Nilsson D.-E. 2009 The evolution of eyes and visually guided behaviour. Phil. Trans. R. Soc. B 364, 2833–2847 (doi:10.1098/rstb.2009.0083) 19720648 10.1098/rstb.2009.0083 PMC2781862

[RSTB20090102C51] Nordström K. Larsson T. A. Larhammar D. 2004 Extensive duplications of phototransduction genes in early vertebrate evolution correlate with block (chromosome) duplications. Genomics 83, 852–872 (doi:10.1016/j.ygeno.2003.11.008) 15081115 10.1016/j.ygeno.2003.11.008

[RSTB20090102C52] Okano T. Kojima D. Fukada Y. Shichida Y. Yoshizawa T. 1992 Primary structures of chicken cone visual pigments: vertebrate rhodopsins have evolved out of cone visual pigments. Proc. Natl Acad. Sci. USA 89, 5932–5936 (doi:10.1073/pnas.89.13.5932) 1385866 10.1073/pnas.89.13.5932 PMC402112

[RSTB20090102C53] Okano T. Yoshizawa T. Fukada Y. 1994 Pinopsin is a chicken pineal photoreceptive molecule. Nature 372, 94–97 (doi:10.1038/372094a0) 7969427 10.1038/372094a0

[RSTB20090102C54] Peirson S. N. Halford S. Foster R. G. 2009 The evolution of irradiance detection: melanopsin and the non-visual opsins. Phil. Trans. R. Soc. B 364, 2849–2865 (doi:10.1098/rstb.2009.0050) 19720649 10.1098/rstb.2009.0050 PMC2781857

[RSTB20090102C55] Quesada A. Genis-Galvez J. M. 1985 Morphological and structural study of Landolt's club in the chick retina. J. Morphol. 184, 205–214 (doi:10.1002/jmor.1051840210) 3989868 10.1002/jmor.1051840210

[RSTB20090102C56] Ready D. F. Tepass U. 2004 Crumbs-dependent epithelial organization in retinal morphogenesis and disease. In Recent advances in human biology. *Vol. 10*. Photoreceptor cell biology and inherited retinal degenerations (ed. Williams D. S.), pp. 7–27 River Edge, NJ: World Scientific Publishers (ISBN 978-981-238-864-3, eISBN 978-981-256-175-6)

[RSTB20090102C57] Rieke F. Baylor D. A. 2000 Origin and functional impact of dark noise in retinal cones. Neuron 26, 181–186 (doi:10.1016/S0896-6273(00)81148-4) 10798402 10.1016/s0896-6273(00)81148-4

[RSTB20090102C58] Scheerer P. Park J. H. Hildebrand P. W. Kim Y. J. Krauss N. Choe H. W. Hofmann K. P. Ernst O. P. 2008 Crystal structure of opsin in its G-protein-interacting conformation. Nature 455, 497–502 (doi:10.1038/nature07330) 18818650 10.1038/nature07330

[RSTB20090102C59] Shichida Y. Matsuyama T. 2009 Evolution of opsins and phototransduction. Phil. Trans. R. Soc. B, 364, 2881–2895 (doi:10.1098/rstb.2009.0051) 19720651 10.1098/rstb.2009.0051 PMC2781858

[RSTB20090102C60] Solovei I. Kreysing M. Lanctôt C. Kösem S. Peichl L. Cremer T. Guck J. Joffe B. 2009 Nuclear architecture of rod photoreceptor cells adapts to vision in mammalian evolution. Cell 137, 356–368 (doi:10.1016/j.cell.2009.01.052) 19379699 10.1016/j.cell.2009.01.052

[RSTB20090102C61] Strettoi E. Dacheux R. F. Raviola E. 1990 Synaptic connections of rod bipolar cells in the inner plexiform layer of the rabbit retina. J. Comp. Neurol. 295, 449–466 (doi:10.1002/cne.902950309) 2351763 10.1002/cne.902950309

[RSTB20090102C62] Strettoi E. Raviola E. Dacheux R. F. 1992 Synaptic connections of the narrow-field, bistratified rod amacrine cell (AII) in the rabbit retina. J. Comp. Neurol. 325, 152–168 (doi:10.1002/cne.903250203) 1460111 10.1002/cne.903250203

[RSTB20090102C63] Suga H. Schmid V. Gehring W. J. 2008 Evolution and functional diversity of jellyfish opsins. Curr. Biol. 18, 51–55 (doi:10.1016/j.cub.2007.11.059) 18160295 10.1016/j.cub.2007.11.059

[RSTB20090102C64] Takahashi Y. Hisatomi O. Sakakibara S. Tokunaga F. Tsukahara Y. 2001 Distribution of blue-sensitive photoreceptors in amphibian retinas. FEBS Lett. 501, 151–166 (doi:10.1016/S0014-5793(01)02632-1) 11470275 10.1016/s0014-5793(01)02632-1

[RSTB20090102C65] Terakita A. 2005 The opsins. Genome Biol. 6, 213 (doi:10.1186/gb-2005-6-3-213) 15774036 10.1186/gb-2005-6-3-213 PMC1088937

[RSTB20090102C66] Terakita A. Koyanagi M. Tsukamoto H. Yamashita T. Miyata T. Shichida Y. 2004 Counterion displacement in the molecular evolution of the rhodopsin family. Nat. Struct. Mol. Biol. 11, 284–289 (doi:10.1038/nsmb731) 14981504 10.1038/nsmb731

[RSTB20090102C67] Thomas M. M. Lamb T. D. 1999 Light adaptation and dark adaptation of human rod photoreceptors measured from the *a*-wave of the electroretinogram. J. Physiol. 518, 479–496 (doi:10.1111/j.1469-7793.1999.0479p.x) 10381594 10.1111/j.1469-7793.1999.0479p.x PMC2269441

[RSTB20090102C68] Travis G. H. Golczak M. Moise A. R. Palczewski K. 2007 Diseases caused by defects in the visual cycle: retinoids as potential therapeutic agents. Annu. Rev. Pharmacol. Toxicol. 47, 469–512 (doi:10.1146/annurev.pharmtox.47.120505.105225) 16968212 10.1146/annurev.pharmtox.47.120505.105225 PMC2442882

[RSTB20090102C69] Tsuda M. Kusakabe T. Iwamoto H. Horie T. Nakashima Y. Nakagawa M. Okunou K. 2003 Origin of the vertebrate visual cycle: II. Visual cycle proteins are localized in whole brain including photoreceptor cells of a primitive chordate. Vision Res. 43, 3045–3053 (doi:10.1016/j.visres.2003.09.012) 14611940 10.1016/j.visres.2003.09.012

[RSTB20090102C70] Turner D. L. Cepko C. L. 1987 A common progenitor for neurons and glia persists in rat retina late in development. Nature 328, 131–136 (doi:10.1038/328131a0) 3600789 10.1038/328131a0

[RSTB20090102C71] Vaney D. I. Young H. M. Gynther I. C. 1991 The rod circuit in the rabbit retina. Vis. Neurosci. 7, 141–154 (doi:10.1017/S0952523800011019) 1931798 10.1017/s0952523800011019

[RSTB20090102C72] Wässle H. 2004 Parallel processing in the mammalian retina. Nat. Rev. Neurosci. 5, 1–11 10.1038/nrn1497 15378035

[RSTB20090102C73] Wässle H. Yamashita M. Greferath U. Grünert U. Müller F. 1991 The rod bipolar cell of the mammalian retina. Vis. Neurosci. 7, 99–112 (doi:10.1017/S095252380001097X) 1718403 10.1017/s095252380001097x

[RSTB20090102C74] Wetts R. Fraser S. E. 1988 Multipotent precursors can give rise to all major cell types of the frog retina. Science 239, 1142–1145 (doi:10.1126/science.2449732) 2449732 10.1126/science.2449732

[RSTB20090102C75] Yamasu T. Yoshida M. 1976 Fine structure of complex ocelli of a cubomedusan, *Tamoya bursaria* Haekel. Cell Tissue Res. 170, 325–339 (doi:10.1007/BF00219415) 8210 10.1007/BF00219415

[RSTB20090102C76] Yan E. C. Y. Kazmi M. A. Ganim Z. Hou J. M. Pan D. H. Chang B. S. W. Sakmar T. P. Mathies R. A. 2003 Retinal counterion switch in the photoactivation of the G protein-coupled receptor rhodopsin. Proc. Natl Acad. Sci. USA 100, 9262–9267 (doi:10.1073/pnas.1531970100) 12835420 10.1073/pnas.1531970100 PMC170906

[RSTB20090102C77] Yokoyama S. 2000 Molecular evolution of vertebrate visual pigments. Prog. Ret. Eye Res. 19, 385–419 (doi:10.1016/S1350-9462(00)00002-1) 10.1016/s1350-9462(00)00002-1 10785616

